# The relationship between methodological quality and the use of retracted publications in evidence syntheses

**DOI:** 10.1186/s13643-023-02316-z

**Published:** 2023-09-20

**Authors:** Caitlin J. Bakker, Nicole Theis-Mahon, Sarah Jane Brown, Maurice P. Zeegers

**Affiliations:** 1 Dr. John Archer Library and Archives, University of Regina, 3737 Wascana Parkway, Regina, SK S4S 0A2 Canada; 2 Department of Epidemiology, School for Nutrition and Translational Research in Metabolisms, Care and Health Research Institute, Maastricht University Medical Center +, PO Box 616, 6200 MD Maastricht, The Netherlands; 3 Health Sciences Libraries, University of Minnesota Twin Cities, Phillips-Wangensteen Building, 516 Delaware Street SE, Minneapolis, MN 55455 USA

**Keywords:** Retraction of publication, Scientific misconduct, Systematic reviews, Research integrity, Publication ethics

## Abstract

**Background:**

Evidence syntheses cite retracted publications. However, citation is not necessarily endorsement, as authors may be criticizing or refuting its findings. We investigated the sentiment of these citations—whether they were critical or supportive—and associations with the methodological quality of the evidence synthesis, reason for the retraction, and time between publication and retraction.

**Methods:**

Using a sample of 286 evidence syntheses containing 324 citations to retracted publications in the field of pharmacy, we used AMSTAR-2 to assess methodological quality. We used scite.ai and a human screener to determine citation sentiment. We conducted a Pearson’s chi-square test to assess associations between citation sentiment, methodological quality, and reason for retraction, and one-way ANOVAs to investigate association between time, methodological quality, and citation sentiment.

**Results:**

Almost 70% of the evidence syntheses in our sample were of critically low quality. We found that these critically low-quality evidence syntheses were more associated with positive statements while high-quality evidence syntheses were more associated with negative citation of retracted publications. In our sample of 324 citations, 20.4% of citations to retracted publications noted that the publication had been retracted.

**Conclusion:**

The association between high-quality evidence syntheses and recognition of a publication’s retracted status may indicate that best practices are sufficient. However, the volume of critically low-quality evidence syntheses ultimately perpetuates the citation of retracted publications with no indication of their retracted status. Strengthening journal requirements around the quality of evidence syntheses may lessen the inappropriate citation of retracted publications.

## Introduction

Scientific discovery is not a linear process; it requires verification, replication, and correction. This correction may come in the form of retraction of scientific publications and could be necessitated by a range of reasons, from errors in methodology or reporting, ethical concerns or authorship disputes, or data falsification and fabrication. Retractions are becoming an increasingly common corrective mechanism, growing from an average of 240 retractions per year in the 2000s to over 1500 retractions per year in the subsequent decade [1]. However, to say that science has been corrected would indicate that researchers understand that a publication has been retracted and the context surrounding the retraction. The continued use of retracted publications can be considered a proxy for the efficacy of this corrective mechanism. If a retracted publication is treated as if it is valid and used as the groundwork for future research, this may indicate that the process of retraction is ineffective.

Citation of retracted publications is not inherently problematic, as citation may not necessarily be an endorsement. The examination of the meaning and function of citations is well-established, with formal approaches dating back to the early 1960s [2, 3]. Since then, two primary schools of thought have been established: the social constructivist, in which scholars use citations for persuasive purposes, and the normative, in which scholars use citation as a means of giving credit and acknowledgement of achievement [4, 5]. Numerous taxonomies have been created [6–10]. While these taxonomies vary in degrees of granularity, structure, and terminology, the majority consider the interpretation of the scholar’s positioning of the work they were citing (i.e., whether an endorsement or a critique), whether the cited material is being treated as something core to the scholar’s work or is a more limited level of engagement.

However, taxonomies which are based upon researcher motivations have faced criticism. As Tahamtan and Bornmann note, the choice to cite a particular document may be motivated by factors beyond the control of the author, including external influences such as the recommendations of editors or peer reviewers, or characteristics specific to the author, such as academic background and topic knowledge [11]. Furthermore, attempts to uncover researcher motivation in their citation decisions are often problematic and subject to the limitations of any data gathered through self-report [12].

Emerging approaches to citation sentiment analysis, which are influenced by computational linguistics, natural language processing, and machine learning, attempt to employ broader categories of positive, negative, and neutral [13]. These approaches do not attempt to determine the author’s intention, but instead consider the citation in the context of the manuscript and describe how the citation is operating in the scholarly literature [14].

Previous research has shown that retracted publications indeed continue to be cited by other researchers and are often cited without any recognition of the retraction status of the research [15–17]. Such citation is particularly problematic when these citations are occurring in systematic reviews and other types of evidence syntheses. Evidence syntheses are often positioned at the pinnacle of the evidence hierarchy and are intended to be a rigorous examination of the totality of the evidence on a particular topic, with the potential to provide the basis for decisions in policy and practice [18–20]. Evidence syntheses are the preferred research method underpinning patient care decisions, decisions on health insurance provision and coverage, health system policy decisions, and more. A lack of effective evidence synthesis has been directly tied to delays in implementing effective treatment options for patients, perpetuation of ineffective and harmful treatments, unnecessary risks to patients and research participants, and inefficient use of research funding and resources [21]. However, for evidence synthesis to be effective in improving policy and practice, it must be based upon sound science. In contrast, the majority of retracted publications are retracted due to misconduct [22–24]. This may include compromised peer review processes, data falsification, image manipulation, and fabricated results.

Despite the importance of evidence synthesis and its function as a rigorous examination of the evidence, they are not immune to the potential impact of retracted publications. Kataoka et al. examined 587 systematic reviews and clinical practice guidelines (CPGs) that cited retracted randomized controlled trials (RCTs) [25]. They found that of the 252 systematic reviews and CPGs that cited previously retracted publications, 67% made no mention of the retracted status of the RCT. Of the 335 systematic reviews and CPGs that cited RCTs that were later retracted, 3% were later corrected and 11% excluded the RCT at the time, either due to concerns about the study or inclusion criteria. Eighty percent incorporated the retracted RCT and were not subsequently corrected. This reinforces Avenell et al.’s previous findings that of 68 evidence syntheses that cited retracted publications—including 13 of which would have their findings changed by the removal of the retracted publication from the analysis—only one undertook reassessment [26].

When there is potentially flawed research incorporated into evidence syntheses, it may raise questions about the conclusions of these syntheses and the rigor of the methods that produced them. Findings on the impact of retracted publications on the statistical findings of meta-analyses have varied significantly. One recent study found that in their sample of 229 meta-analyses cited retracted publications, only 21 indicated that the retracted publication had been retracted; however, removing the data associated with those retracted publications from the pooled summaries of meta-analyses did not significantly alter the results [15]. In contrast, other studies show substantial influence of retracted publications. A reanalysis of 22 meta-analyses removing data associated with a retracted publication altered the results of over half of the meta-analyses [27], and the exclusion of two publications from a meta-analysis of ivermectin and COVID-19 invalidated that meta-analysis’s previous finding of decreased mortality [28].

While the impact of retractions on the findings of specific meta-analyses may vary, treating retracted publications as valid research in the context of evidence syntheses is problematic. Not only does it have the potential to influence findings of the synthesis, but it also perpetuates the use of the retracted publication and its associated findings and undermines the function of retraction as a corrective mechanism. While previous research affirms that retracted publications continue to be cited as valid in evidence syntheses, the methodological quality of these evidence syntheses and its relationship to the use of retracted publications has not been explored. To the best of our knowledge, no research has assessed the methodological quality of evidence syntheses citing retracted publications.

We sought to address the following research questions:

1. What is the methodological quality of evidence syntheses citing retracted publications?
2. Are evidence syntheses citing retracted publications indicating that the publications have been retracted?
3. Is there an association between the methodological quality of the evidence syntheses and whether they indicate that the publications have been retracted?
4. Is there a relationship between the length of time between publication and retraction and an association with a retracted publication being indicated as such or the methodological quality of the evidence synthesis?

## Methods

A previous research project identified evidence syntheses that cited retracted publications in pharmacy [29]. This project was based on retracted publications identified through the Retraction Watch Database [30]. From a list of retracted publications, we created a subset of 1396 retracted publications in the fields of pharmacology, toxicology, and drug design. These fields were selected to reflect the breadth of the field of pharmacy, which was chosen as it extends from bench research to clinical care and has potential impact on other healthcare specialties, such as surgery, anesthesia, and family medicine. Known item searching was then conducted in Web of Science and Scopus to retrieve all citing publications. 32,559 publications which cited these retracted items were retrieved. Titles and abstracts of citing publications were then screened by two independent reviewers using Rayyan to confirm that they were evidence syntheses. This was then followed by full-text screening phase, which was completed by two independent researchers. Any discrepancies were resolved through consensus.

Publications were excluded if they were not evidence syntheses, which was defined as systematic reviews, scoping reviews, rapid reviews, meta-analyses, or clinical practice guidelines. Publications were also excluded if they were subsequently retracted. Evidence syntheses published in languages other than English were excluded due to the linguistic nuance necessary to assess citation sentiment. No limitations were placed on year of publication.

**Fig. 1:**
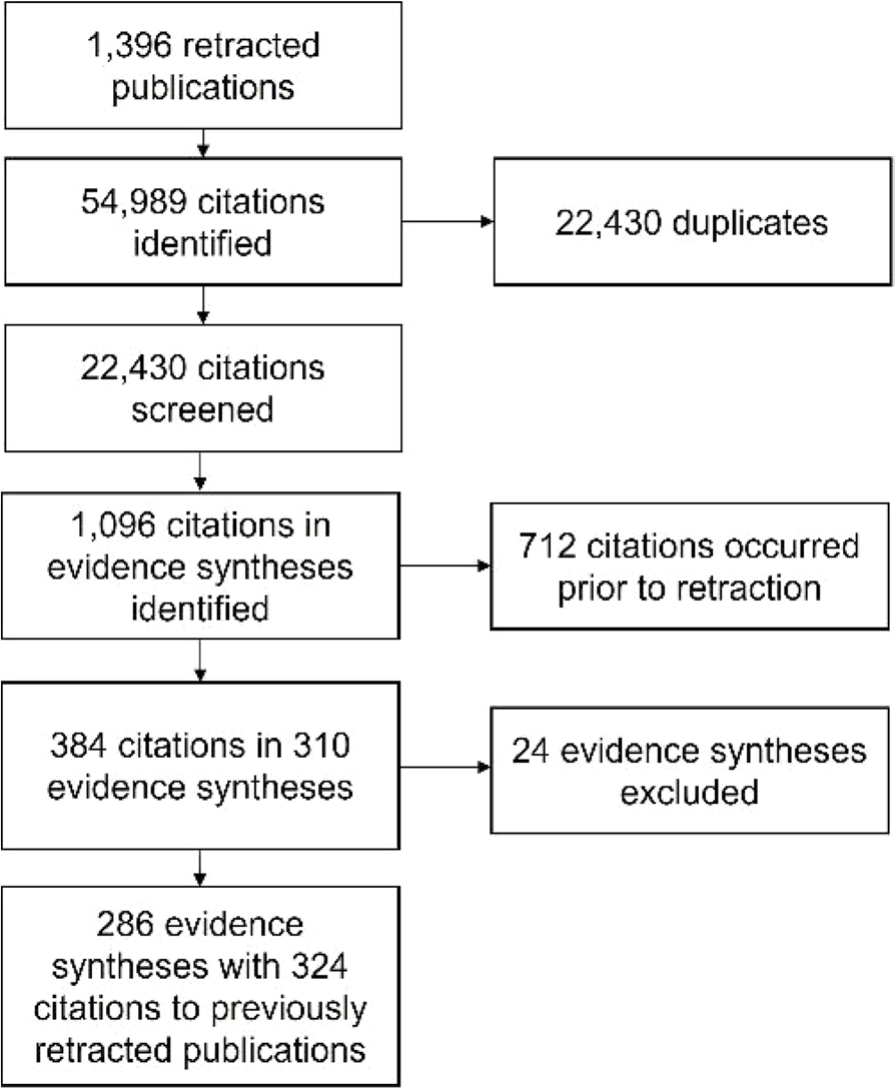
Identification of evidence syntheses citing retracted publications

This previous project identified 1096 citations to retracted publications in evidence syntheses, including 712 that occurred prior to retraction and 384 that occurred after the publication had been retracted. This research project isolated 384 citations occurring in 310 evidence syntheses. From the original set of 310 evidence syntheses, 24 were excluded because the evidence synthesis was not in English (*n* = 9), the evidence synthesis was subsequently retracted (*n* = 4), the citation to the retracted article could not be found in the full text or the references (*n* = 6), the item was a duplicate or co-publication (*n* = 4), or it was determined not to be an evidence synthesis (*n* = 1). Our sample included 286 evidence syntheses containing 324 citations to retracted research. The process of identifying these evidence syntheses is shown in Fig. 1.

We utilized previous mapping of the Retraction Watch Database’s reason for retraction to a modified version of Bar Ilan and Halevi’s taxonomy of reasons for retraction [31]. Bar Ilan and Halevi’s taxonomy includes three broad classifications: (1) scientific distortion, including data falsification and errors; (2) ethical misconduct, including plagiarism and IRB issues; and (3) administrative error, such as a journal erroneously publishing the wrong version of an article. We further subdivided scientific distortion to scientific distortion—falsification and manipulation, which refers to instances of intentional distortion, and scientific distortion—concerns or errors, in which scientific distortion occurred but intention was not proven, such as an unintentional error in data collection or analysis. Bar Ilan and Halevi posited that scientific distortion was the most problematic of the classifications, as the publication’s findings may be unsound and could subsequently lead to misdirected research in the future or false conclusions. While ethical misconduct is troubling, it does not necessarily invalidate the findings of the research.

Data collection was completed using a Qualtrics form. We established agreement in our assessment by having all researchers independently code 5% of the total sample. Upon assessment of the sample, we found near perfect agreement and subsequently the remaining evidence syntheses were assessed by one independent reviewer. Methodological quality of the evidence synthesis was assessed using the AMSTAR 2 criteria [32]. The AMSTAR 2 checklist is designed to aid in the assessment of the methodological quality of systematic reviews. The 16 questions in AMSTAR 2 relate to 16 domains or potential weaknesses, 7 of which are critical and 9 of which are non-critical. A critical weakness is one that is thought to have potential impact on the overall validity of findings, while a non-critical weakness is one that is indicative of methodological quality but may not impact overall validity. The AMSTAR 2 criteria result in one of four overall ratings: high quality, moderate quality, low quality, and critically low quality. A high-quality review has no more than one non-critical weakness, a moderate-quality review has more than one non-critical weakness but no critical weaknesses, a low-quality review has one critical weakness, and a critically low-quality review has more than one critical weakness.

We used scite.ai. to capture the sentiment of the citation, by which we mean the reason the authors were citing the paper. Scite is a web-based tool that uses machine learning algorithms to identify which articles have cited which studies, and whether those citations were supporting, mentioning, or contrasting [33]. Scite refers to this as a classification of rhetorical function, intending to describe whether the citing paper is supporting or contrasting claims made in the paper it is citing. Scite’s terminology has been aligned with the more commonly used positive, negative, and neutral categories prominent in citation sentiment analysis for clarity. We used a second Qualtrics form to capture scite’s assessment of the sentiment of the citation. Researchers independently noted their agreement or disagreement with the assessment. In the case where the researcher disagreed with the scite’s assessment, the researcher’s interpretation was recorded. Assessments were reviewed by all researchers to ensure consistency and agreement. The findings of this assessment are outlined elsewhere [34].

To assess for associations between the categorical variables of citation sentiment and methodological quality and reason for retraction, we conducted a Pearson’s chi-square test with a simulated *p* value based on 2000 replicates. To investigate association between the mean time between publication and retraction for publications grouped by methodological quality and grouped by citation sentiment, we conducted one-way ANOVAs and post hoc Tukey tests. All analyses were conducted in R 4.1.1.

## Results

Of the 286 syntheses citing retracted publications, the majority (199, 69.6%) were found to be of critically low quality, 49 (17.1%) were found to be of low quality, 21 (7.3%) were found to be of moderate quality, and 17 (5.9%) were found to be of high quality according to AMSTAR 2. Details of the findings of the methodological assessment are outlined in Table 1.

**Table 1:** Assessment of methodological quality according to AMSTAR 2 criteria

	Yes *n* (%)	Partial yes *n* (%)	No *n* (%)	No meta-analysis conducted *n* (%)
***Critical domains/weaknesses***				
Did the report of the review contain an explicit statement that the review methods were established prior to the conduct of the review and did the report justify any significant deviations from the protocol?	63 (22)	11 (3.8)	212 (74.1)	N/A
Did the review authors use a comprehensive literature search strategy?	30 (10.5)	136 (47.6)	120 (42)	N/A
Did the review authors provide a list of excluded studies and justify the exclusions?	162 (56.6)	N/A	124 (43.4)	N/A
Did the review authors use a satisfactory technique for assessing the risk of bias (RoB) in individual studies that were included in the review?	172 (60.1)	N/A	114 (39.9)	N/A
If meta-analysis was performed did the review authors use appropriate methods for statistical combination of results?	166 (58)	N/A	24 (8.4)	96 (33.6)
If they performed quantitative synthesis did the review authors carry out an adequate investigation of publication bias (small study bias) and discuss its likely impact on the results of the review?	135 (47.2)	N/A	55 (19.2)	96 (33.6)
***Non-critical domains/weaknesses***				
Did the research questions and inclusion criteria for the review include the components of PICO?	260 (90.9)	N/A	26 (9.1)	N/A
Did the review authors explain their selection of the study designs for inclusion in the review?	229 (80.1)	N/A	57 (19.9)	N/A
Did the review authors perform study selection in duplicate?	162 (56.6)	N/A	124 (43.4)	N/A
Did the review authors perform data extraction in duplicate?	164 (57.3)	N/A	122 (42.7)	N/A
Did the review authors describe the included studies in adequate detail?	92 (32.2)	123 (43)	71 (24.8)	N/A
Did the review authors report on the sources of funding for the studies included in the review?	40 (14)	N/A	246 (86)	N/A
If meta-analysis was performed, did the review authors assess the potential impact of RoB in individual studies on the results of the meta-analysis or other evidence synthesis?	117 (40.9)	N/A	73 (25.5)	96 (33.6)
Did the review authors account for RoB in individual studies when interpreting/discussing the results of the review?	141 (49.3)	N/A	145 (50.7)	N/A
Did the review authors provide a satisfactory explanation for, and discussion of, any heterogeneity observed in the results of the review?	180 (62.9)	N/A	106 (37.1)	N/A
Did the review authors report any potential sources of conflict of interest, including any funding they received for conducting the review?	247 (86.4)	N/A	39 (13.6)	N/A

Of the 324 citations to retracted publications, the majority (140, 43.2%) were positive while 118 (36.4%) were neutral and 66 (20.4%) were negative. We found statistically significant associations between citation sentiment and methodological quality (*X*^2^: 44.39, df = NA, *p* = 0.0005). High-quality studies were more associated with negative statements and were less associated with positive statements, while critically low-quality studies were more associated with positive statements and less associated with negative statements. We also found a statistically significant association between citation sentiment and reason for the article’s retraction (*X*^2^: 28.405, df = NA, *p* value = 0.001). Articles retracted for scientific distortion due to falsification and manipulation were more associated with negative citations and were less associated with positive citations, while articles retracted due to ethical misconduct were more associated with positive citations. These findings are detailed in Table 2.

**Table 2:** Factors associated with citation sentiment

	Positive *n* (%)	Neutral *n* (%)	Negative *n* (%)	*P* value
***Methodological quality***				
High quality	1 (0.3)	7 (2.2)	14 (4.3)	0.0005
Moderate quality	6 (1.9)	10 (3.1)	10 (3.1)	
Low quality	22 (6.8)	20 (6.2)	14 (4.3)	
Critically low quality	111 (34.3)	81 (25)	28 (8.6)	
***Reason for retraction***				
Scientific distortion—manipulation or falsification	18 (5.6)	29 (9)	26 (8)	0.001
Scientific distortion—concerns or errors	53 (16.4)	53 (16.4)	26 (8)	
Ethical misconduct	61 (18.8)	30 (9.3)	13 (4)	
Administrative error	4 (1.2)	4 (1.2)	0 (0)	
Unknown	4 (1.2)	2 (0.6)	1 (0.3)	

Within our sample, the mean time between publication and retraction was 1646 days or 4.5 years. There was no statistically significant association between timing and the quality of the evidence synthesis (*p* = 0.25). There was a statistically significant association between timing and citation sentiment (*p* = 0.0386, *f* value = 3.287). On average, positive citations had a period between publication and retraction that was 564 days (1.56 years) shorter than negative citations. Findings are detailed in Table 3. A one-way ANOVA revealed no statistically significant interactions between the reason for retraction and the citation sentiment when considering the time between publication and retraction.

**Table 3:** Days between publication and retraction and its association with methodological quality and citation sentiment

	Days mean (sd)	*P* value
***Methodological quality***		
High quality	2266 (2013)	0.25
Moderate quality	1704 (1853)	
Low quality	1627 (1415)	
Critically low quality	1570 (1478)	
***Citation sentiment***		
Positive	1412 (1281)	0.039
Neutral	1718 (1789)	
Negative	1976 (1526)	

## Discussion

This study found that the vast majority of evidence synthesis studies citing retracted publications were of critically low quality with only 7% being of high quality. These findings are in line with other research investigating the quality of evidence syntheses in general. One study investigating the quality of systematic reviews in dentistry found that 68% of the reviews were of critically low quality and none was of high quality [35], while another in urology found that only 4.2% of reviews were of high quality [36]. We found that high- and moderate-quality evidence syntheses were associated with negative citations to retracted publications, while syntheses of critically low quality were associated with positive citations and did not include negative citations. The association between methodological quality and the ways in which retracted publications were cited may indicate that existing best practices in conducting evidence syntheses are effective in addressing the challenge of retracted publications.

There is increasingly robust documentation on best practices in identifying retractions which has become available [37], as well as technological innovations such as the integration of Retraction Watch data into EndNote and Zotero [38, 39]. The recent integration of Retraction Watch data by Third Iron into their LibKey and Browzine products has the potential to further these efforts by alerting library users of a publication’s retracted status [40]. Surfacing this information at the point of discovery rather than positioning it as an additional verification step has the potential to further help researchers identify flawed research and avoid integrating it into their own work. Such technical solutions, particularly where they leverage third-party, vendor agnostic data, can have a tremendous impact in improving the clarity and consistency with which the retracted status of publications are communicated.

While this is heartening, enthusiasm is somewhat tempered when considering the large number of poor-quality evidence syntheses found. It is difficult to determine why low-quality evidence syntheses are undertaken and continue to be published. Previous research has found that the rate at which evidence syntheses are produced has grown dramatically—1930% between 2000 and 2019 [41]—but that the citation and usage of evidence syntheses have decreased over time. Halevi and Pinotti found that as the number of systematic reviews increased, the average number of citations, citations in policy documents, downloads, and views decreased [42]. This could suggest that an exponential increase in published evidence synthesis results in poorer quality studies going unnoticed due to the decrease in citations and views.

Despite the overall low quality of the evidence syntheses in this sample, it is possible that the quality is still being overestimated. AMSTAR 2 has several limitations as a tool for methodological assessment. The assessment of the adequacy of the search may not be sufficient. While the AMSTAR 2 criteria do require that “[k]ey words and/or MESH terms should be reported and the full search strategy available upon request” [32], there is no requirement to consider the appropriateness of the terms (subject terms or keywords alone are appropriate) or the comprehensiveness, structure, or replicability of the search. The PRESS Guidelines provide a significantly more in-depth assessment mechanism with requirements for Boolean operators, subject headings and keywords, spelling variations, and filters [43]. PRISMA-S and PRISMA 2020 Reporting Checklists—both of which were released after AMSTAR 2—provide further tools for assessing the comprehensive reporting of searches [44, 45]. PRISMA-S complements PRISMA 2020 and offers guidelines creating reproducible searches for evidence synthesis. The AMSTAR 2 search criteria may need to be updated to reflect the comprehensiveness and rigor a systematic review search strategy requires.

Previous research on the experience of information professionals engaged in evidence syntheses has found significant challenges in ensuring methodological rigor. Surveys have found that between 28 and 68% of information professionals report challenges with researchers not following appropriate systematic review methodology [46, 47]. While low-quality evidence syntheses are regularly published in a range of disciplines, few journals provide guidance on methodological quality of systematic reviews and meta-analyses in their author guidelines [48]. Strengthening journal requirements around the methodological quality of systematic reviews and meta-analyses may aid in lessening the inappropriate citation of retracted publications.

The majority of citations to retracted publications in these evidence syntheses did not indicate that the publication had been retracted. We found that the majority of our citations were positive, followed by citations that mentioned the retracted publication in passing but did not indicate that it had been retracted. This would indicate that, in our sample, evidence syntheses that cite retracted publications are not identifying that the publication has been retracted. These findings are consistent with previous research, including two studies which independently found that over 94% of citations to retracted publications do not indicate that the publication was retracted [16, 49]. Future research could explore the correlations between citing retracted publications in evidence syntheses and having a librarian or informational professional as a co-author.

We found an association between publications retracted due to ethical misconduct and positive citation, while publications retracted due falsification and manipulation were more associated with negative citations. In the context of Bar-Ilan and Halevi’s taxonomy, this does indicate that the most potentially damaging science is being recognized as such in our sample. However, the continued positive citation of publications previously retracted for ethical misconduct is nevertheless problematic. While retraction is meant to correct the scientific record, it is also intended to disincentivize unethical behavior. While some previous research has found that retracted publications receive fewer citations than their non-retracted counterparts [50], both citation and self-citation of publications continue following retraction [51]. While it is beyond the scope of this project to investigate the associations between the impact on findings and the reason for retraction, this would be a useful area of future research.

Negative citations were associated with a significantly longer time between publication and retraction. This may initially appear counterintuitive, as one might expect that a publication that has a longer time in the scholarly ecosystem without correction would become more entrenched and therefore more likely to accrue positive or neutral citations. However, it should be noted that of the 66 negative citations in our sample, 28 were associated with 4 primary authors, 3 of whom have the dubious distinction of being in the top 5 on Retraction Watch’s Leaderboard [52]. The retractions were generally associated with long-standing ethical and scientific misconduct which spanned multiple years and impacted a cumulative 476 publications. Removal of the 28 publications associated with these 4 researchers reduced the average period between publication and retraction to 1423 days (3.89 years), which does not differ significantly from that of the positive citations (1412 days, 3.86 years). It is possible that the notoriety of these cases and the publicity surrounding these retractions increased the likelihood that they would receive negative citations in comparison to retractions that did not receive as much publicity.

While it is not possible to state conclusively why individuals are citing retracted publications positively based on publication data alone, previous research describes the inconsistency with which the retracted status of publications is displayed. One 2018 study looked at this issue across different bibliographic databases, finding that some platforms display fewer than 5% of retracted publications as retracted [53]. A more recent 2020 white paper reinforced these findings and noted the variability even within a single journal [54]. This inconsistency in the indication of the retracted status of publications has been found in disciplinary journals, including research in emergency medicine and dentistry which found that watermarking of retracted publications ranged from 40 to 57% [55, 56]. While we cannot state that an inconsistent representation perpetuates the citation of retracted publications, it does stand to reason that if publications are not clearly marked as being retracted, a reader would be less likely to realize that the publication had been retracted and would therefore be less likely to reflect that understanding in their own work.

Authors of systematic reviews can play a dual role in both modifying their existing practices and in advocating for clearer and more consistent representation by publishers and aggregators. A recently launched NISO Working Group is developing recommended practices for metadata display and transfer “to improve the dissemination of retraction information and to support consistent, timely transmission of that information to the reader” [57]. Such guidelines may ultimately lead to more consistently and accurately represented retractions. Adoption of these forthcoming guidelines, as well as the development and implementation of retraction policies, should be encouraged.

Evidence syntheses are a powerful tool to identify, appraise, and synthesize scholarship and to improve patient care, accelerate research, and contribute to evidence-based policy. However, for evidence synthesis to perform these functions, it must be based upon sound science. Evidence syntheses that include retracted publications without indication of their retracted status perpetuate the citation and use of those publications and raise questions regarding the rigor of these evidence syntheses and the validity of their findings. As evidence syntheses may impact clinical decision making and policy, the downstream impact of evidence syntheses that incorporate retracted publications, including their incorporation into policy and practice guidelines would be a useful area for future research.

Our study has several limitations. We focus specifically on evidence syntheses in the field of pharmacy due to its breadth and impact. Previous research has established concerns around methodological quality of systematic reviews in a range of fields [35, 36] and has found that publications citing retracted items in multiple disciplines do not indicate that these items have been retracted [16, 17, 49]. However, it is possible that the associations we found between methodological quality and citation sentiment may not be generalizable to other disciplines. Future research could extend this research to other disciplines.

Citation sentiment analysis cannot determine why an author chose to cite a particular article or conversely how they became aware of the retracted status of an article. It also cannot determine whether existing methodological guidance was influential in uncovering the retracted status of an article. Future research should consider how authors become aware of the retractions and which mechanisms and interventions are most effective.

## Conclusion

Science requires continual revision; it is a process of adjusting theories in response to contradictory evidence [58]. However, for that adjustment to occur, this contradictory evidence must be observed. Significant progress has been made in providing guidance to facilitate identification of retracted publications in evidence syntheses. Despite the availability of this guidance, the continued use of retracted publications in evidence syntheses is common and may be a consequence of the prevalence of low-quality evidence syntheses. While ongoing efforts to educate users about this issue are valuable, requirements from journals and publishers, and the adoption of consistent practices by bibliographic databases have the potential to modify researcher behavior, improving the overall quality of evidence syntheses while ensuring that retracted publications are recognized as such.

## Data Availability

Datasets supporting the conclusions of this article as available in the Data Repository for the University of Minnesota, https://hdl.handle.net/11299/241503 [59]. Original raw data used to identify the retracted publications was provided courtesy of the Center for Scientific Integrity’s Retraction Watch Database and is governed by a data use agreement. Retraction Watch data are available by contacting team@retractionwatch.com.

## References

[1] Vuong QH, La VP, Ho MT, Vuong TT, Ho MT (2020). Characteristics of retracted articles based on retraction data from online sources through February 2019. Sci Ed.

[2] Garfield E. What does automation of citation mean? In: Essays of an information scientist. Vol. 1. Philadelphia: ISI Press; 1970. p 98-99.

[3] Garfield E. Can citation indexing be automated? In: Stevens ME, Giuliano VE, Heilprin LB, editors. Statistical Association Methods for Mechanized Documentation, Symposium Proceedings, Washington, 1964. National Bureau of Standards Miscellaneous Publication, vol. 269. US Government Printing Office; 1965. p 189–92.

[4] Cozzens SE (1988). What do citations count?. Scientometrics.

[5] Merton RK. The sociology of science: theoretical and empirical investigations - Robert K. Merton - Google Books. Chicago, IL: University of Chicago Press; 1973. Available from: https://books.google.ca/books?hl=en&lr=&id=zPvcHuUMEMwC&oi=fnd&pg=PR9&ots=x7TLQjc4zT&sig=0cylzmgmSwDjWPmTWdmm78H91XY&redir_esc=y#v=onepage&q&f=false. Cited 2023 May 3.

[6] Brooks TA (1985). Private acts and public objects: an investigation of citer motivations. J Am Soc Inf Sci.

[7] Chubin DE, Moitra SD (1975). Content analysis of references: adjunct or alternative to citation counting?. Soc Stud Sci.

[8] Maricic S, Spaventi J, Pavicic L, Pifat-Mrzljak G (1998). Citation context versus the frequency counts of citation histories. J Am Soc Inf Sci.

[9] Moravcsik MJ, Murugesan P (1975). Some results on the function and quality of citations. Soc Stud Sci.

[10] Teufel S, Siddharthan A, Tidhar D. An annotation scheme for citation function. In: Proceedings of the 7th SIGdial Workshop on Discourse and Dialogue. Sydney, Australia: Association for Computational Linguistics; 2006. 80–7. Available from: https://aclanthology.org/W06-1312. Cited 2023 May 3.

[11] Tahamtan I, Bornmann L (2018). Core elements in the process of citing publications: conceptual overview of the literature. J Informetr.

[12] Wilkinson D, Harries G, Thelwall M, Price L (2003). Motivations for academic web site interlinking: evidence for the Web as a novel source of information on informal scholarly communication. J Inf Sci.

[13] Yousif A, Niu Z, Tarus JK, Ahmad A (2019). A survey on sentiment analysis of scientific citations. Artif Intell Rev.

[14] Bar-Ilan J, Halevi G (2017). Post retraction citations in context: a case study. Scientometrics.

[15] Fanelli D, Wong J, Moher D (2021). What difference might retractions make? An estimate of the potential epistemic cost of retractions on meta-analyses. Account Res.

[16] Hsiao TK, Schneider J (2021). Continued use of retracted papers: temporal trends in citations and (lack of) awareness of retractions shown in citation contexts in biomedicine. Quant Sci Stud.

[17] Schneider J, Ye D, Hill AM, Whitehorn AS (2020). Continued post-retraction citation of a fraudulent clinical trial report, 11 years after it was retracted for falsifying data. Scientometrics.

[18] Campbell DM, Redman S, Rychentnik L, Cooke M, Zwi AB, Jorm L. Increasing the use of evidence in health policy: practice and views of policy makers and researchers. Aust N Z Health Policy. 2009;6(1). Available from: https://www.publish.csiro.au/hp/hp090621. Cited 2022 Aug 20. 10.1186/1743-8462-6-21 PMC2739528 19698186

[19] Gough D, Elbourne D (2002). Systematic research synthesis to inform policy, practice and democratic debate. Soc Policy Soc.

[20] Vogel JP, Oxman AD, Glenton C, Rosenbaum S, Lewin S, Gülmezoglu AM (2013). Policymakers’ and other stakeholders’ perceptions of key considerations for health system decisions and the presentation of evidence to inform those considerations: an international survey. Health Res Policy Syst.

[21] Grimshaw J. A guide to knowledge synthesis. Canadian Institutes of Health Research. 2010. Available from: https://cihr-irsc.gc.ca/e/41382.html. Cited 2023 May 3.

[22] Campos-Varela I, Ruano-Raviña A (2019). Misconduct as the main cause for retraction. A descriptive study of retracted publications and their authors. Gac Sanit.

[23] Fang FC, Steen RG, Casadevall A (2012). Misconduct accounts for the majority of retracted scientific publications. Proc Natl Acad Sci.

[24] Moylan EC, Kowalczuk MK (2016). Why articles are retracted: a retrospective cross-sectional study of retraction notices at BioMed Central. BMJ Open.

[25] Kataoka Y, Banno M, Tsujimoto Y, Ariie T, Taito S, Suzuki T (2022). Retracted randomized controlled trials were cited and not corrected in systematic reviews and clinical practice guidelines. J Clin Epidemiol.

[26] Avenell A, Stewart F, Grey A, Gamble G, Bolland M (2019). An investigation into the impact and implications of published papers from retracted research: systematic search of affected literature. BMJ Open.

[27] Garmendia CA, Nassar Gorra L, Rodriguez AL, Trepka MJ, Veledar E, Madhivanan P (2019). Evaluation of the inclusion of studies identified by the FDA as having falsified data in the results of meta-analyses: the example of the apixaban trials. JAMA Intern Med.

[28] Manu P, Lawrie TA, Dowswell  T, Fordham EJ, Mitchell S, Hill SR, Tham TC, Expression of Concern for Bryant A (2021). Ivermectin for prevention and treatment of COVID-19 infection: a systematic review, meta-analysis, and trial sequential analysis to inform clinical guidelines. Am J Ther.

[29] Brown SJ, Bakker CJ, Theis-Mahon NR (2022). Retracted publications in pharmacy systematic reviews. J Med Libr Assoc.

[30] Center for Scientific Integrity. Retraction watch database.. Available from: http://retractiondatabase.org/RetractionSearch.aspx?. Cited 2022 Jul 21.

[31] Bar-Ilan J, Halevi G (2018). Temporal characteristics of retracted articles. Scientometrics.

[32] Shea BJ, Reeves BC, Wells G, Thuku M, Hamel C, Moran J (2017). AMSTAR 2: a critical appraisal tool for systematic reviews that include randomised or non-randomised studies of healthcare interventions, or both. BMJ.

[33] Nicholson JM, Mordaunt M, Lopez P, Uppala A, Rosati D, Rodrigues NP (2021). Scite: a smart citation index that displays the context of citations and classifies their intent using deep learning. Quant Sci Stud.

[34] Bakker CJ, Theis-Mahon NR, Brown SJ. Evaluating the accuracy of scite, a smart citation index. Hypothesis. 2023;35(2). 10.18060/26528.

[35] Hammel C, Pandis N, Pieper D, Faggion CM (2022). Methodological assessment of systematic reviews of in-vitro dental studies. BMC Med Res Methodol.

[36] Ding M, Soderberg L, Jung JH, Dahm P (2020). Low methodological quality of systematic reviews published in the urological literature (2016–2018). Urology.

[37] Lefebvre C, Glanville J, Briscoe S, Littlewood A, Marshall C, Metzendorf M, et al. Searching for and selecting studies. In: Higgins JPT, Thomas J, Chandler J, Cumpston M, Li T, Page MJ, et al., editors. Cochrane Handbook for Systematic Revies of Interventions version 63. Chichester, UK: John Wiley & Sons, Ltd; 2022. 67–107. Available from: 10.1002/9781119536604.ch4. Cited 2022 Jul 1.

[38] Price G. EndNote adds retraction watch notification integration, similar service available for zotero and papers. Library Journal infoDOCKET. 2021. Available from: https://www.infodocket.com/2021/11/10/endnote-adds-retractionwatch-integration-similar-service-also-available-from-zotero/. Cited 2022 Jul 1.

[39] Stillman D. Retracted item notifications with Retraction Watch integration. Zotero. 2019. Available from: https://www.zotero.org/blog/retracted-item-notifications/. Cited 2022 Jul 27.

[40] About Article Retractions. Third Iron.. Available from: https://support.thirdiron.com/knowledgebase/articles/1976565-about-article-retractions. Cited 2022 Sep 26.

[41] Hoffmann F, Allers K, Rombey T, Helbach J, Hoffmann A, Mathes T (2021). Nearly 80 systematic reviews were published each day: observational study on trends in epidemiology and reporting over the years 2000–2019. J Clin Epidemiol.

[42] Halevi G, Pinotti R (2020). Systematic reviews: characteristics and impact. Publ Res Q.

[43] McGowan J, Sampson M, Salzwedel DM, Cogo E, Foerster V, Lefebvre C (2016). PRESS peer review of electronic search strategies: 2015 guideline statement. J Clin Epidemiol.

[44] Page MJ, McKenzie JE, Bossuyt PM, Boutron I, Hoffmann TC, Mulrow CD (2021). The PRISMA 2020 statement: an updated guideline for reporting systematic reviews. BMJ.

[45] Rethlefsen ML, Kirtley S, Waffenschmidt S, Ayala AP, Moher D, PRISMA-S Group (2021). PRISMA-S: an extension to the PRISMA Statement for Reporting Literature Searches in Systematic Reviews. Syst Rev.

[46] Nicholson J, McCrillis A, Williams JD (2017). Collaboration challenges in systematic reviews: a survey of health sciences librarians. J Med Libr Assoc JMLA.

[47] Schvaneveldt N, Stellrecht EM (2021). Assessing the roles and challenges of librarians in dental systematic and scoping reviews. J Med Libr Assoc JMLA.

[48] Goldberg J, Boyce LM, Soudant C, Godwin K (2022). Assessing journal author guidelines for systematic reviews and meta-analyses: findings from an institutional sample. J Med Libr Assoc.

[49] Theis-Mahon NR, Bakker CJ (2020). The continued citation of retracted publications in dentistry. J Med Libr Assoc.

[50] Lu SF, Jin GZ, Uzzi B, Jones B (2013). The retraction penalty: evidence from the Web of Science. Sci Rep.

[51] Madlock-Brown CR, Eichmann D (2015). The (lack of) impact of retraction on citation networks. Sci Eng Ethics.

[52] Center for Scientific Integrity. The retraction watch leaderboard. Retraction Watch. 2015. Available from: https://retractionwatch.com/the-retraction-watch-leaderboard/. Cited 2022 Jul 11.

[53] Bakker CJ, Riegelman A (2018). Retracted publications in mental health literature: discovery across bibliographic platforms. J Librariansh Sch Commun..

[54] Suelzer EM, Deal J, Hanus K, Ruggeri BE, Witkowski E (2021). Challenges in identifying the retracted status of an article. JAMA Netw Open.

[55] Chauvin A, De Villelongue C, Pateron D, Yordanov Y (2019). A systematic review of retracted publications in emergency medicine. Eur J Emerg Med.

[56] Faggion CM, Ware RS, Bakas N, Wasiak J (2018). An analysis of retractions of dental publications. J Dent.

[57] National Information Standards Organization. NISO voting members approve work on recommended practice for retracted research. 2021. Available from: https://www.niso.org/press-releases/2021/09/niso-voting-members-approve-work-recommended-practice-retracted-research. Cited 2022 Jul 27.

[58] Quine WV (1951). Two dogmas of empiricism. Philos Rev.

[59] Bakker C, Theis-Mahon N, Brown SJ. Data underlying (the relationship between methodological quality and the use of retracted publications in evidence syntheses). 2022. Available from: http://conservancy.umn.edu/handle/11299/241503. Cited 2022 Nov 25. 10.1186/s13643-023-02316-z PMC10512544 37730590

